# Association of genetic ancestry with HER2, GRB7 AND estrogen receptor expression among Colombian women with breast cancer

**DOI:** 10.3389/fonc.2022.989761

**Published:** 2022-12-22

**Authors:** Laura Rey-Vargas, Lina María Bejarano-Rivera, Juan Carlos Mejia-Henao, Luz F. Sua, Jhon Faustino Bastidas-Andrade, Carlos Andrés Ossa, Luz Dary Gutiérrez-Castañeda, Laura Fejerman, María Carolina Sanabria-Salas, Silvia J. Serrano-Gómez

**Affiliations:** ^1^ Cancer Biology Research Group, National Cancer Institute of Colombia, Bogotá, Colombia; ^2^ Doctoral Program in Biological Sciences, Pontificia Universidad Javeriana, Bogotá, Colombia; ^3^ Oncological Pathology Research Group, National Cancer Institute of Colombia, Bogotá, Colombia; ^4^ Department of Pathology and Laboratory Medicine, Fundación Valle del Lili, and Faculty of Health Sciences, Universidad ICESI, Cali, Colombia; ^5^ Oncology Unit, Fundación San Pedro Hospital, Pasto, Colombia; ^6^ Cancer Institute, Las Américas Clinic, Medellín, Colombia; ^7^ Research Institute, Group of Basic Sciences in Health (CBS), Fundación Universitaria de Ciencias de la Salud (FUCS), Bogotá, Colombia; ^8^ Department of Public Health Sciences and Comprehensive Cancer Center, University of California Davis, Davis, CA, United States; ^9^ Research support and follow-up group, National Cancer Institute of Colombia, Bogotá, Colombia

**Keywords:** breast neoplasms, American native continental ancestry, receptor ErbB-2, GRB7 adaptor protein, estrogen receptor

## Abstract

**Background:**

Our previous study reported higher mRNA levels of the human epidermal growth factor receptor 2 (HER2)-amplicon genes *ERBB2* and *GRB7* in estrogen receptor (ER)-positive breast cancer patients with relatively high Indigenous American (IA) ancestry from Colombia. Even though the protein expression of HER2 and GRB7 is highly correlated, they may also express independently, an event that could change the patients’ prognosis. In this study, we aimed to explore the differences in ER, HER2 and GRB7 protein expression according to genetic ancestry, to further assess the clinical implications of this association.

**Methods:**

We estimated genetic ancestry from non-tumoral breast tissue DNA and assessed tumoral protein expression of ER, HER2, and GRB7 by immunohistochemistry in a cohort of Colombian patients from different health institutions. We used binomial and multinomial logistic regression models to test the association between genetic ancestry and protein expression. Kaplan-Meier and log-rank tests were used to evaluate the effect of HER2/GRB7 co-expression on patients’ survival.

**Results:**

Our results show that patients with higher IA ancestry have higher odds of having HER2+/GRB7- breast tumors, compared to the HER2-/GRB7- subtype, and this association seems to be stronger among ER-positive tumors (ER+/HER2+/GRB7-: OR=3.04, 95% CI, 1.47-6.37, p<0.05). However, in the multivariate model this association was attenuated (OR=1.80, 95% CI, 0.72-4.44, p=0.19). On the other hand, it was observed that having a higher European ancestry patients presented lower odds of ER+/HER2+/GRB7- breast tumors, this association remained significant in the multivariate model (OR=0.36, 95% CI, 0.13 - 0.93, p= 0.0395). The survival analysis according to HER2/GRB7 co-expression did not show statistically significant differences in the overall survival and recurrence-free survival.

**Conclusions:**

Our results suggest that Colombian patients with higher IA ancestry and a lower European fraction have higher odds of ER+/HER2+/GRB7- tumors compared to ER+/HER2-/GRB7- disease. However, this association does not seem to be associated with patients’ overall or recurrence-free survival.

## Introduction

Breast cancer is the malignancy with the highest incidence (47.8 per 100,000) and mortality rates among women worldwide (13.6 per 100,000) (1). At the molecular level, it is a heterogeneous disease that has been classified into four major intrinsic subtypes (luminal A, luminal B, human epidermal growth factor receptor 2 (HER2)-enriched, and basal-like or triple negative) based on tumor’s gene expression profiles (2). Each of these subtypes has a different clinical prognosis. Breast cancer luminal subtypes, characterized by the positive expression of estrogen receptor (ER) and progesterone receptor (PR), often show a well to moderately differentiated phenotype, a low cellular proliferation index, and are associated with a relatively good survival probability. On the other hand, basal-like and HER2-enriched subtypes, both negative for hormone receptors expression, often present an aggressive phenotype; these patients are frequently diagnosed at higher clinical stages with nodal involvement and larger tumors (3, 4).

Differences in the prevalence of breast tumor subtypes by population group have been widely described (5–7). Epidemiologic studies have consistently reported that non-Hispanic White (NHW) women have a higher prevalence of luminal A disease, whilst African American/Black (AA/B) and Hispanic/Latina women have a higher prevalence of basal-like and HER2-enriched subtypes (7–9). Possible contributors to these disparities include differences in the presentation of several reproductive (parity, duration of lactation and age at first birth) and socioeconomic risk factors (socioeconomic status and health insurance) among populations (10–15). Genetic ancestry proportion has also been linked to differences in clinical-pathological characteristics of breast cancer among these population groups, including differences in the distribution of breast cancer subtypes (16–19). A recent study conducted in Peruvian women, a population with relatively high average of Indigenous American (IA) ancestry proportion, reported a 20% increase in the odds of developing HER2-positive breast tumors per every 10% increment in the IA ancestry fraction (OR=1.20, 95% CI, 1.07-1.35, *p=*0.001) (20), and this association was especially strong for ER-negative tumors. We have also shown that Colombian women with higher IA ancestry (>36%) expressed higher mRNA levels of the *ERBB2* gene, although in this study, only ER-positive tumors were analyzed (21).

*ERBB2* is located in the 17q12 region, at the so-called HER2 amplicon, and it is usually co-amplified with other genes such as *GRB7* (22). Additionally, HER2/GRB7 co-expression has been associated with resistance to anti-HER2 treatments and with poor prognosis (23–25). It has also been reported that HER2 may express independently from GRB7, and this event might confer a different prognosis (22). It is still unclear whether IA ancestry is associated with the co-expression of both proteins, and what it is the linkage between this association with ER expression. Additionally, the clinical implications of HER2/GRB7 co-expression in the Colombian population have not yet been explored. Therefore, in this study we tested differences in ER, HER2 and GRB7 protein expression according to genetic ancestry, as well as the association of different combinations of expression of these proteins with breast cancer survival in Colombian women.

## Materials and methods

### Sample selection

We revised the clinical-pathological data from breast cancer patients diagnosed between 2013 and 2015 at the Colombian National Cancer Institute (NCI) in Bogotá D.C, a national reference center for cancer treatment that admits patients from all country regions. A cohort of 361 patients were selected according to the following inclusion criteria: 1) histologically confirmed diagnosis of invasive ductal carcinoma (IDC), 2) availability of formalin-fixed paraffin-embedded (FFPE) tissue blocks that contained at least 10% of tumor content from mastectomies or breast-conserving surgeries, and 3) availability of FFPE blocks with no-tumor content. Patients with *in situ* carcinoma were excluded. In order to enrich the population sample with patients from different regions and different genetic ancestry proportions, we invited other institutions to participate in the study. Breast cancer patients diagnosed at the San Pedro Hospital (SPH) (n=55), the Fundación Valle de Lili (FVL) University Hospital (n=73), and Las Américas Clinic (LAC) (n=28), were also included under the same selection criteria. Biospecimens from a total of 517 patients were included in this study. This research was approved by the ethics committee from all four institutions, and according to the Colombian laws, it was considered that no informed consent was required.

### Immunohistochemistry

Immunohistochemistry (IHC) assays were performed on 3 µm thick sections from a single FFPE surgery block with the highest tumor content, using monoclonal antibodies for ER (clone SP1 Roche 05278406001), PR (clone 1E2 Roche 05278392001), HER2 (clone 4B5 Roche 05278368001), Ki67 (clone 30-9 Roche 05278384001) and GRB7 (A-12 sc-376069, Santa Cruz Biotechnology), using the Roche Benchmark XT automated slide preparation system (Roche Ltd., Switzerland). Positive controls were included and 3,3′ diaminobenzidine (DAB) was used as the chromogen.

A single pathologist analyzed the IHC expression of the ER, PR, HER2 and Ki67 biomarkers. Status of hormone receptors was considered positive when they exceeded 1% of nuclear staining in tumor cells. HER2 evaluation followed the recommendations of the American Society of Clinical Oncology (ASCO)/College of American Pathologists (CAP) guideline (26) and was defined as: positive (3+) for complete and intense circumferential membrane within >10% of tumor cells; ambiguous (2+) for incomplete and/or weak/moderate circumferential membrane staining within > 10% of tumor cells, or complete membrane staining but within ≤10% of tumor cells; negative (1+) for incomplete faint membrane staining within >10% of tumor cells; and negative (0+) for absence of staining. HER2 ambiguous (2+) cases with no confirmatory fluorescence *in situ* hybridization (FISH)/chromogenic *in situ* hybridization (CISH) test were excluded from the analysis. GRB7 expression was assessed as the percentage of tumor cells with positive membrane/cytoplasmic staining. Cases with ≥10% of GRB7 membrane/cytoplasmic staining were defined as positive, while the remaining cases (<10% staining) were defined as negative.

### Genetic ancestry estimation

DNA was extracted from non-tumor paraffin blocks using the AllPrep DNA/RNA FFPE kit (Qiagen, Inc., Valencia, CA, USA) and the RecoverAll Total Nucleic Acid for FFPE kit (Invitrogen, Carlsbad, CA, USA) following the manufacturer’s protocol. Nucleic acid concentration was quantified by NanoDrop ND1000 Spectrophotometer (Thermo Scientific, Wilmington, USA). A panel of 106 Single Nucleotide Polymorphisms (SNPs) previously validated as Ancestry Informative Markers (AIMs) (21) were genotyped at the University of Minnesota Genomics Center, using the Sequenom technology. SNPs with a call rate <90% or that deviated from Hardy-Weinberg equilibrium were removed from the analysis, leaving 87 SNPs for individual genetic ancestry estimation. A total of 495 samples were genotyped and 381 remained after excluding samples with a genotype call rate <85% (NCI= 308/361; FVL= 29/73; SPH= 44/55; LAC= 0/28).

We genotyped 10 duplicate pairs and the overall discordance rate was 0. Quality control of the genotyped data was performed in PLINK 1.9 (27), and the software Admixture 1.3 (28) was used under an admixture model (k=3) to estimate IA, European and African ancestry proportions.

### Statistical analysis

All statistical analyses were performed using the RStudio software version 1.2.5019. Continuous variables presented a non-normal distribution and were reported as medians and interquartile ranges (IQR). Categorical variables were summarized as absolute and relative frequencies. We applied a Kruskal–Wallis test to assess differences in genetic ancestry fractions according to the status of ER, HER2 and GRB7, and tumor subtype (ER/HER2); and a Chi-square (*X^2^*) test to assess differences in categorical variables. Unknown and not classifiable categories were not included in statistical analysis.

We used a univariate logistic regression model to evaluate the association between the expression of ER, HER2 and GRB7 per every 25% increase in genetic ancestry fractions. A univariate multinomial logistic regression model was used to assess the association between genetic ancestry and: (1) Breast cancer subtypes categorized by ER/HER2 expression and (2) ER/HER2/GRB7 co-expression status. For the multivariate logistic regression model, we included potential cofounding variables such as health institution, clinical stage, and age at diagnosis.

We evaluated differences in overall survival (OS) and disease-free survival (DFS) according to HER2/GRB7 co-expression status using the Kaplan-Meier and log rank test. OS was calculated from the date of diagnosis to the date of death or last follow-up. DFS was calculated from the date of surgery to the date of the first recurrence (local, regional, or distant relapse) or last follow-up. Differences were considered statistically significant if p<0.05.

## Results

### Patients’ characteristics

Patients’ clinical-pathological characteristics according to health institution are described in **Table 1**. We observed statistically significant differences in all variables evaluated. At the NCI, we observed a higher percentage of patients diagnosed with breast cancer over the age of 50 (77.0%) with late clinical stages (III/IV: 45.7%) and positive histological invasion (51.5%). Most of the patients from the FVL presented poorly differentiated tumors (Scarff-Bloom Richardson III: 49.2%), had lymph node involvement (76.9%), and 42.5% had deceased at the time of the study. A higher percentage of patients from the SPH presented moderately differentiated tumors (Scarff-Bloom Richardson II: 50.9%) and had the highest proportion of recurrence (32.7%) among all health institutions. Overall, patients from the LAC presented favorable clinical-pathological features such as early clinical stages (I: 60.7%), negative histological invasion (75.0%), and no clinical recurrence at the time of the study.

**Table 1:** Clinical-pathological characteristics of patients by health institution.

	NCI (n = 361)	FVL (n = 73)	SPH (n = 55)	LAC (n = 28)	*p* value
	N (%)	N (%)	N (%)	N (%)	
Age of diagnosis					
≤50 years	83 (23.0)	27 (37.0)	25 (45.5)	6 (21.4)	<0.001^a^
>50 years	278 (77.0)	40 (54.8)	30 (54.5)	21 (75.0)	
Unknown	0 (0.0)	6 (8.2)	0 (0.0)	1 (3.6)	
AJCC Clinical stage					
I (I, Ia, Ib)	40 (11.1)	10 (13.7)	4 (7.3)	17 (60.7)	<0.001^a^
II (IIa, IIb)	156 (43.2)	29 (39.7)	30 (54.5)	10 (35.7)	
III (IIIa, IIIb, IIIc)/IV	165 (45.7)	28 (38.4)	18 (32.7)	1 (3.6)	
Unknown	0 (0.0)	6 (8.2)	3 (5.5)	0 (0.0)	
Scarff-Bloom Richardson					
I	30 (8.3)	9 (13.8)	21 (38.2)	8 (28.6)	<0.001^a^
II	192 (53.2)	24 (36.9)	28 (50.9)	19 (67.9)	
III	138 (38.2)	32 (49.2)	4 (7.3)	1 (3.6)	
Unknown	1 (0.3)	0 (0.0)	2 (3.6)	0 (0.0)	
Tumor size					
≤ 20 mm	96 (26.6)	26 (35.6)	18 (32.7)	0 (0.0)	0.206^a^
> 20 mm	253 (70.1)	46 (63.0)	28 (50.9)	1 (3.6)	
Unknown	12 (3.3)	1 (1.4)	9 (16.4)	27 (96.4)	
Histological invasion					
Negative	139 (38.5)	28 (38.4)	26 (47.3)	21 (75.0)	0.005^a^
Positive	186 (51.5)	45 (61.6)	25 (45.5)	7 (25.0)	
Unknown	36 (10.0)	0 (0.0)	4 (7.3)	0 (0.0)	
Lymph node involvement					
Negative	188 (52.1)	12 (23.1)	11 (25.0)	1 (10.0)	<0.001^a^
Positive	173 (47.9)	40 (76.9)	33 (75.0)	9 (90.0)	
Unknown	0 (0.0)	21 (28.8)	11 (20.0)	18 (64.3)	
Ki67 status					
High (≥20%)	213 (59.0)	29 (39.7)	31 (56.4)	4 (14.3)	<0.001^a^
Low (<20%)	148 (41.0)	44 (60.3)	23 (41.8)	24 (85.7)	
Unknown	0 (0.0)	0 (0.0)	1 (1.8)	0 (0.0)	
ER/HER2 tumor subtype					
ER+/HER2-	204 (56.5)	50 (68.5)	26 (47.3)	23 (82.1)	<0.001^a^
ER+/HER2+	34 (9.4)	8 (11.0)	11 (20.0)	3 (10.7)	
ER-/HER2+	21 (5.8)	7 (9.6)	9 (16.4)	1 (3.6)	
ER-/HER2-	55 (15.2)	8 (11.0)	5 (9.1)	1 (3.6)	
Not classifiable	47 (13.0)	0 (0.0)	4 (7.3)	0 (0.0)	
Clinical recurrence					
Negative	247 (68.4)	24 (32.9)	30 (54.5)	28 (100.0)	<0.001^a^
Positive	87 (24.1)	22 (30.1)	18 (32.7)	0 (0.0)	
Unknown	27 (7.5)	27 (37.0)	7 (12.7)	0 (0.0)	
Vital state					
Alive	277 (76.7)	41 (56.2)	32 (58.2)	28 (100.0)	<0.001^a^
Deceased	83 (23.0)	31 (42.5)	6 (10.9)	0 (0.0)	
Unknown	1 (0.3)	1 (1.4)	17 (30.9)	0 (0.0)	
Genetic ancestry (median [IQR])					
European ancestry fraction	0.51 [0.43, 0.59]	0.48 [0.33, 0.62]	0.36 [0.26, 0.44]	Not available	<0.001^b^
IA ancestry fraction	0.40 [0.32, 0.48]	0.32 [0.19, 0.42]	0.58 [0.51, 0.69]	Not available	<0.001^b^
African ancestry fraction	0.07 [0.03, 0.12]	0.12 [0.03, 0.21]	0.03 [0.00, 0.08]	Not available	<0.001^b^

NCI, National Cancer Institute; FVL, Fundación Valle de Lili; SPH, San Pedro Hospital; LAC, Las Américas Clinic; ER, estrogen receptor; IA, Indigenous American; IQR, interquartile range.

Statistical tests: ^a^Fisher’s exact test, ^b^Kruskal-Wallis

Unknown and not classifiable categories were not included in the statistical analysis.

Statistically significant differences between health institutions were observed for ER/HER2 expression (*p*<0.001) and Ki67 status (*p*<0.001). The ER+/HER2- subtype was the most prevalent in all patients, although it was especially frequent among patients from the LAC (82.1%). SPH had a higher number of patients with HER2+ tumors (ER+/HER2+: 20%, ER-/HER2+: 16.4%) and ER-/HER2- tumors were more frequently observed at the NCI (15.2%) compared to other health institutions.

### Genetic ancestry distribution according to ER, HER2 and GRB7 breast tumor expression

**Figure 1:**
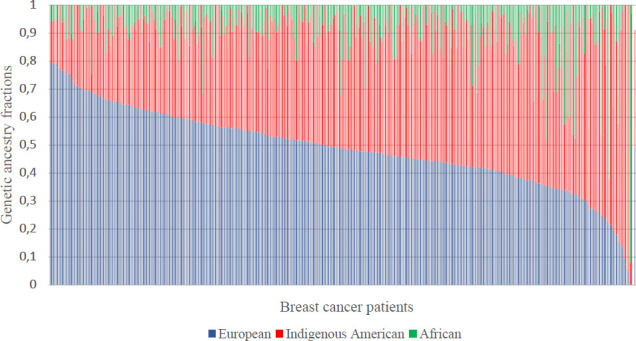
Genetic ancestry distribution for 381 breast cancer patients from Columbia. Each patient is represented by a vertical bar at the x-axis.

Genetic ancestry data was available for 73.7% of the cases (381/517). The average genetic ancestry proportions for the European, IA, and African components were 48.9%, 42.1%, and 8.9%, respectively (**Figure 1**). We observed statistically significant differences in genetic ancestry fractions by health institution (**Table 1**). European and IA ancestry components were significantly higher in the NCI (0.51) and the SPH (0.58), respectively, whereas African ancestry was higher in the FVL (0.12).

We analyzed differences in genetic ancestry by ER, HER2 and GRB7 status (**Table 2**). We observed that both HER2 and GRB7 positive cases presented a higher IA ancestry, compared to the negative group (HER2 positive (3+): 0.44 vs. negative (0+/1+): 0.40, *p*=0.003; GRB7-positive: 0.43 vs negative: 0.41, *p*=0.019). HER2-negative cases also showed a significantly higher median of European ancestry, compared to the HER2-positive group (0.49 vs 0.44, respectively, *p*=0.024). Regarding the African component, no statistically significant differences were observed in HER2 and GRB7 expression by this fraction. Similarly, ER expression also did not show any statistically significant changes by genetic ancestry.

**Table 2:** Differences of genetic ancestry fractions according to the status of ER, HER2 and GRB7, and their co-expression.

	N=381	European (median [IQR])	*p* value	IA (median [IQR])	*p* value	African (median [IQR])	*p* value
ER status							
Positive	293	0.48 [0.42, 0.59]	0.651	0.41 [0.32, 0.51]	0.396	0.07 [0.03, 0.12]	0.536
Negative	88	0.50 [0.41, 0.57]		0.42 [0.34, 0.51]		0.06 [0.02, 0.11]	
HER2 status*							
Positive (3+)	66	0.44 [0.34, 0.57]	0.024	0.44 [0.37, 0.57]	0.003	0.06 [0.03, 0.13]	0.362
Negative (0+/1+)	273	0.49 [0.42, 0.58]		0.40 [0.32, 0.50]		0.07 [0.03, 0.12]	
GRB7 status							
Positive	52	0.47 [0.42, 0.56]	0.256	0.43 [0.40, 0.54]	0.019	0.06 [0.02, 0.10]	0.276
Negative	329	0.49 [0.42, 0.59]		0.41 [0.32, 0.50]		0.07 [0.03, 0.12]	
ER/HER2 subtype*							
ER+/HER2-	218	0.49 [0.42, 0.59]	0.053	0.40 [0.31, 0.50]	0.023	0.07 [0.03, 0.12]	0.452
ER+/HER2+	39	0.43 [0.34, 0.53]		0.45 [0.40, 0.55]		0.06 [0.03, 0.15]	
ER-/HER2+	27	0.50 [0.38, 0.60]		0.43 [0.34, 0.57]		0.05 [0.01, 0.09]	
ER-/HER2-	55	0.51 [0.41, 0.56]		0.42 [0.33, 0.50]		0.06 [0.03, 0.12]	
ER/HER2/GRB7 co-expression^+^							
ER+/HER2-/GRB7-	217	0.49 [0.43, 0.59]	0.009	0.40 [0.31, 0.49]	0.020	0.07 [0.03, 0.12]	0.558
ER+/HER2+/GRB7-	15	0.35 [0.27, 0.42]		0.55 [0.42, 0.63]		0.06 [0.03, 0.13]	
ER+/HER2+/GRB7+	24	0.47 [0.42, 0.56]		0.43 [0.39, 0.51]		0.07 [0.03, 0.15]	
ER-/HER2+/GRB7+	20	0.45 [0.36, 0.57]		0.48 [0.40, 0.58]		0.05 [0.01, 0.07]	
ER-/HER2+/GRB7-	7	0.60 [0.47, 0.62]		0.35 [0.28, 0.47]		0.07 [0.04, 0.11]	
ER-/HER2-/GRB7-	54	0.51 [ 0.41, 0.56]		0.42 [0.33, 0.50]		0.07 [0.03, 0.12]	

IA, Indigenous American; IQR, interquartile range.

*HER2 equivocal (2+) cases with no confirmatory result (n=42) were excluded from the analysis.

^+^HER2-/GRB7+ tumors (n=2) were excluded from the analysis because of low representation.

Statistical test: Kruskal-Wallis.

The co-expression analysis of ER/HER2 showed a higher median of IA ancestry in ER+/HER2+ tumors compared to ER+/HER2- group (0.45 vs. 0.40, *p=*0.023). Interestingly, when GRB7 was included in the co-expression analysis, we observed the highest IA ancestry fraction in the ER+/HER2+/GRB7- group (0.55 vs. 0.40, *p*=0.02), and the lowest European ancestry values (0.35 vs. 0.49, *p*=0.009), compared to the ER+/HER2-/GRB7- group. The co-expression analysis (ER/HER2/GRB7) did not show any statistically significant differences by African ancestry (**Table 2**).

### Association of genetic ancestry with ER/HER2/GRB7 breast tumor expression

We assessed the association between genetic ancestry and the expression of ER, HER2, GRB7, and their co-expression in a multivariable model. This analysis showed that every 25% increase in the IA ancestry fraction led to a 1.89 increase in the odds of having HER2-positive breast tumors (OR=1.89, 95% CI, 1.22–2.94, *p=*0.0043); on the contrary, higher European ancestry was found associated with lower odds of HER2-positive breast tumors (OR=0.58, 95% CI, 0.36 – 0.91, *p=* 0.0204). After adjusting for potential covariates, the association between IA and European ancestry with HER2 expression was no longer statistically significant (**Table 3** and **Supplementary Table S1**). On the other hand, no statistically significant associations were found between African ancestry and the expression of these proteins (**Supplementary Table S2**).

**Table 3:** Association per every 25% increase in IA ancestry with ER/HER2/GRB7 expression.

	Univariate		Multivariate	
	OR (95% CI)	*p* value	OR (95% CI)	*p* value
ER status				
Negative	1.00		1.00	
Positive	0.87 (0.58-1.39)	0.5065	1.02 (0.63 - 1.66)	0.9180
HER2 status*				
Negative (0+/1+)	1.00		1.00	
Positive (3+)	1.89 (1.22 - 2.94)	0.0043	1.48 (0.89 - 2.46)	0.1289
GRB7 status				
Negative	1.00		1.00	
Positive	1.59 (0.98 - 2.55)	0.0566	1.39 (0.78 - 2.44)	0.2559
ER/HER2 subtype				
ER+/HER2-	1.00		1.00	
ER+/HER2+	1.93 (1.13 - 3.32)	0.0154	1.61 (0.84 - 3.09)	0.1480
ER-/HER2+	1.72 (0.92 - 3.16)	0.0795	1.18 (0.50 - 2.67)	0.6808
ER-/HER2-	1.04 (0.63 - 1.71)	0.8528	1.07 (0.58 - 1.92)	0.8144
ER/HER2/GRB7 co-expression^+^				
ER+/HER2-/GRB7-	1.00		1.00	
ER+/HER2+/GRB7-	3.04 (1.47 - 6.37)	0.00245	1.80 (0.72 - 4.44)	0.1950
ER+/HER2+/GRB7+	1.31 (0.64 - 2.58)	0.436	1.36 (0.56 - 3.20)	0.477
ER-/HER2+/GRB7+	2.14 (1.07 - 4.22)	0.0279	1.43 (0.52 - 3.78)	0.473
ER-/HER2+/GRB7-	0.88 (0.21 - 2.84)	0.8510	0.82 (0.13 - 3.63)	0.809
ER-/HER2-/GRB7-	1.05 (0.63 - 1.73)	0.8294	1.06 (0.58 - 1.91)	0.8317

OR, odd ratio; CI, confidence interval; IA, Indigenous American.

*HER2 equivocal (2+) cases with no confirmatory result (n=42) were excluded from the analysis.

^+^HER2-/GRB7+ tumors (n=2) were excluded from the analysis because of low representation.

The multivariate model was adjusted by health institution, age of diagnosis and clinical stage.

Furthermore, we observed that higher levels of IA ancestry and a lower European ancestry fraction was associated with higher odds of ER+/HER2+ breast tumors (IA ancestry fraction: OR= 1.93, 95% CI, 1.13-3.32, *p=*0.0154; European ancestry fraction: OR= 0.46, 95% CI, 0.26-0.82, *p=* 0.00882). When GRB7 was included in the co-expression analysis (ER/HER2/GRB7), a stronger association was observed between both IA and European ancestry fractions with ER+/HER2+/GRB7- tumors (IA ancestry fraction: OR= 3.04, 95% CI, 1.47 – 6.37, *p*=0.00245; European ancestry fraction: OR= 0.23, 95% CI, 0.1-0.52, *p*=0.000448) (**Table 3** and **Supplementary Table S1**). However, in the multivariate model the reported association between genetic ancestry and the ER+/HER2+/GRB7- subtype remained statistically significant only for the European component (OR= 0.36, 95% CI, 0.13-0.93, *p*= 0.0395) (**Supplementary Table S1**). Regarding the African component, the co-expression analysis for ER+/HER2+/GRB7- did not show any statistically significant associations with this ancestry fraction (**Supplementary Table S2**).

### Differences in clinical–pathological characteristics and outcomes by HER2/GRB7 co-expression

We explored differences in the presentation of clinical-pathological features among breast cancer patients according to HER2/GRB7 status (**Supplementary Table S3**). This analysis showed that HER2+/GRB7- and HER2+/GRB7+ patients were more frequently diagnosed under the age of 50 (39.5% and 39.3% vs. 25.7%, respectively, *p*=0.036) and presented higher proliferation rates (Ki67 status ≥20%: 71.1% and 87.5% vs. 45.5%, respectively, *p*<0.001), compared to HER2-/GRB7- patients. However, we observed a higher frequency of less differentiated tumors only for patients with the HER2/GRB7 co-expression, compared to both HER2+/GRB7- and HER2-/GRB7- patients (Scarff-Bloom Richardson III: 53.6% vs. 36.8% and 30.5%, respectively, *p*=0.007). No statistically significant differences were found for OS and RFS by the co-expression status (**Supplementary Figures S1**).

## Discussion

We aimed to assess the association between genetic ancestry and the protein expression of ER, HER2, and GRB7, along with its clinical implications in Colombian patients with breast cancer. In an effort to have a better representation of the three main ancestry fractions in Colombia (European, IA, and African), breast cancer patients from four different health institutions around the country were included. As a result, we observed a great heterogeneity in the patient’s clinicopathological features according to health institution. This is a problem hospital-based studies in Latin-America often face as a consequence of the different treatment protocols used for cancer management in each health institution, but also the profile of people that live in each specific area. We observed a higher proportion of patients diagnosed at earlier stages in some institutions, while in others, patients presented worse disease outcomes, seen as higher recurrences. This might be closely related to patient’s socioeconomic factors who attend each institution, like insurance regime (subsidized and contributory) and access to health services (29–31). All of these are challenges that need to be addressed in further studies in Latin-American countries (32).

Differences in genetic ancestry were also observed according to health institution. Patients that came from health centers in the Andean region, such as the NCI, showed the highest European ancestry, whereas patients from the FVL and SPH had higher proportions of African and IA ancestry components respectively. This is expected as the FVL is located in Cali, a city in the Colombian Pacific region, where a strong influx of African population happened during colonization (33, 34), explaining the highest fraction of African ancestry in patients from this health institution. On the other hand, the SPH, located in the city of Pasto, has a strong IA influence, and receives more patients from rural areas, explaining the high fraction of IA ancestry in this region (33).

As previously reported, our findings suggest that breast cancer patients with a higher IA ancestry and a lower European component might have an increased risk of developing HER2-positive tumors. As for GRB7, a gene commonly co-expressed with HER2 due to their proximity on chromosome 17, position q12, we only found it associated with IA and European ancestry when we analyzed its expression alone. However, when we analyzed it in a HER2/GRB7 co-expression model, the association was observed only for HER2+/GRB7- tumors, suggesting that the association found with genetic ancestry is mostly driven by the HER2 status. As for the hormone receptor, no statistically significant associations were observed between ER expression alone and genetic ancestry. However, when we analyzed it in a model together with HER2 and GRB7 co-expression, we observed a stronger association with IA and European ancestry among ER-positive tumors. Based on these results, we hypothesize that this protein might have a potential role in the association between genetic ancestry and the protein expression of HER2. Further studies are needed to keep exploring these results.

Population-based studies have showed that Hispanic/Latina women from United States with breast cancer, have a higher proportion of HER2-positive tumors when compared to NHW patients (7, 35, 36). Results from two hospital-based studies from Latin America have consistently reported an association between IA ancestry fraction and HER2 expression (20, 21). The first one, conducted in Colombian breast cancer patients with luminal subtypes reported a higher expression of the *ERBB2* gene in patients with higher IA ancestry (>36%) (21), while the second one, conducted in a large population of Peruvian women, consistently reported a higher risk of developing HER2-positive tumors per every 10% increment in the IA ancestry (20). Although we did not find the association with the IA ancestry component in the multivariate model, we still observed a consistent trend. On the other hand, this association was still significant in the multivariate model for the European component, which might be due to the higher contribution of this particular ancestry component among the Colombian population (16, 33). A positive correlation between ER-negative tumors and a higher African ancestry has been widely explored and documented before (19, 37), however, in this study we did not replicate these previous findings, possibly due to the lower representation of this specific component among our Colombian sample population.

So far, the genetic basis of this association remains unclear, however, as a possible explanatory mechanism, it has been proposed that there might be genetic variants located within ancestry-specific genomic regions, with the ability to regulate gene expression. These genetic variants are known as expression Quantitative Trait Loci (eQTLs) (38, 39). This hypothesis is supported by a large study across 33 cancer types from the Cancer Genome Atlas Project (TCGA), where they analyzed germline variant data of 9,899 cases, and reported the presence of ancestry-specific predisposition variants that were associated with an altered expression of the affected genes (e.g., *BRCA2* in samples of African ancestry) (40). Moreover, a genome-wide association study conducted in Latinas did find a genetic variant (rs140068132) that is mostly present in populations with IA ancestry, and is associated with a lower risk of ER-negative breast tumors (41, 42). Overall, these latter studies support the hypothesis that genetic ancestry can impact breast cancer phenotype. Further studies are still needed to explore the molecular mechanisms behind this association and to identify potential population-specific eQTLs that predispose Latina women to develop HER2-positive breast tumors.

Ours is the first study in Latin-American women with breast cancer to assess clinical prognosis according to the co-expression of these proteins. ER and HER2 are well-known biomarkers in breast cancer, widely used for prognosis assessment and to guide treatment protocols (43, 44). GRB7, on the other side, has been less explored in the context of breast cancer. Studies that have assessed its expression in tumor samples, suggest the role of GRB7 as an adaptor protein that binds to tyrosine kinase receptors like HER2, to amplify its signal and mediate the activation of several downstream proteins involved in cell migration and survival pathways (45). It has been shown that tumors with HER2/GRB7 co-expression present even higher nuclear grades and lower survival rates, compared with tumors that only express HER2 (46, 47). However, we did not find any significant differences in the OS and DFS according to the co-expression status, suggesting that GRB7 might not impact the prognosis of breast cancer patients. The introduction of anti-HER2 therapies such as trastuzumab and pertuzumab to treatment schemes for HER2-positive breast tumors have significantly improved the patients’ prognosis (48). This could also be related with the fact that no statistically significant differences in the patients’ survival were found by HER2/GRB7 co-expression status.

During this study, we encountered several limitations. The first and most important one was the heterogeneity of the population included by health institution in terms of clinical-pathological characteristics but also by genetic ancestry. As has been reported before, genetic ancestry not only reflects the genetic profile derived from our ancestors, but is also an indirect reflector of other non-genetic exposures related to lifestyle and environmental risk factors associated with human behavior around populations (18, 49–51). It is possible that such factors can impact the tumor phenotype and the course of the disease, acting as cofounding variables. Another limitation was the small sample size, especially when we evaluated ER/HER2/GRB7 co-expression groups, which may have reduced the study’s statistical power, and limited the opportunity to find biological associations. Additionally, we had a low representation of HER2-positive tumors in our sample. We highlight the need for population-based cancer registries in Latin-America to avoid the effects of unmeasured environmental exposure factors on research studies conducted in undeveloped countries (52).

In conclusion, our results suggest that Colombian patients with higher IA ancestry and a lower European component might have higher odds of developing breast luminal tumors with HER2 overexpression but no GRB7, compared to other subtypes. However, this association does not seem to have an impact on the patients’ overall or recurrence-free survival. We highlight the need to conduct new investigations from population-based cancer studies in Latin America to confirm this association and to explore the genetic basis of these findings. This is an important matter as nowadays the healthcare system is moving towards a more personalized medicine model, and it is crucial to keep gathering as much scientific evidence as possible of the potential effects of genetic ancestry on breast cancer phenotype so that, in the future, this variable may contribute to the decision-making process on patients’ disease management.

## Data availability statement

The original contributions presented in the study are included in the article/**Supplementary Material**. Further inquiries can be directed to the corresponding author.

## Ethics statement

The studies involving human participants were reviewed and approved by Ethics Committee of the National Cancer Institute of Colombia. Written informed consent for participation was not required for this study in accordance with the national legislation and the institutional requirements

## Author contributions

The concept of the study was conceived by SS-G and MS-S. LR-V, LB-R, JM-H, LS, JB-A, CO, and LG-C contributed to data collection. Data analysis and interpretation were performed by LR-V, LB-R, LF, MS-S and SS-G. LR-V wrote the manuscript in collaboration with SS-G. All authors contributed to the article and approved the submitted version.
