# Supplementary material for: Association of genetic ancestry with HER2, GRB7 AND estrogen receptor expression among Colombian women with breast cancer
Source: Front Oncol. 2022 Dec 22;12:989761. doi: 10.3389/fonc.2022.989761 (PMC9815522; doi:10.3389/fonc.2022.989761)
Supplement: Supplementary file 1 [file DataSheet_1.docx]

**Supplementary material**

**Supplementary Table S1.** Association per every 25% increase in European ancestry with ER, HER2 and GRB7 defined groups.

|  | **Univariate** | | **Multivariate** | |
| --- | --- | --- | --- | --- |
|  | **OR (95% CI)** | ***p* value** | **OR (95% CI)** | ***p* value** |
| **ER status** | | | | |
| Negative | 1.00 |  | 1.00 |  |
| Positive | 0.90 (0.59 - 1.38) | 0.6429 | 1.04 (0.63 - 1.73) | 0.8683 |
| **HER2 status*** | | | | |
| Negative (0+/1+) | 1.00 |  | 1.00 |  |
| Positive (3+) | 0.58 (0.36 - 0.91) | 0.0204 | 0.74 (0.44 - 1.25) | 0.264 |
| **GRB7 status** | | | | |
| Negative | 1.00 |  | 1.00 |  |
| Positive | 0.77 (0.47 - 1.29) | 0.3307 | 0.93 (0.52 - 1.67) | 0.8068 |
| **ER/HER2 subtype** | | | | |
| ER+/HER2- | 1.00 |  | 1.00 |  |
| ER+/HER2+ | 0.46 (0.26 - 0.82) | 0.00882 | 0.51 (0.26 - 0.99) | 0.0499 |
| ER-/HER2+ | 0.77 (0.39 - 1.55) | 0.4591 | 1.31 (0.55 - 3.27) | 0.5477 |
| ER-/HER2- | 0.85 (0.50 - 1.44) | 0.5533 | 0.80 (0.43 - 1.49) | 0.486 |
| **ER/HER2/GRB7 co-expression^+^** | | | | |
| ER+/HER2-/GRB7- | 1.00 |  | 1.00 |  |
| ER+/HER2+/GRB7- | 0.23 (0.1 - 0.52) | 0.000448 | 0.36 (0.13 - 0.93) | 0.0395 |
| ER+/HER2+/GRB7+ | 0.78 (0.38 - 1.66) | 0.5219 | 0.73 (0.31 - 1.72) | 0.467 |
| ER-/HER2+/GRB7+ | 0.63 (0.3 - 1.38) | 0.241 | 1.19 (0.43 - 3.44) | 0.739 |
| ER-/HER2+/GRB7- | 1.39 (0.38 - 5.94) | 0.6401 | 1.40 (0.28 - 7.84) | 0.680 |
| ER-/HER2-/GRB7- | 0.84 (0.49 - 1.42) | 0.5148 | 0.80 (0.43 - 1.48) | 0.4755 |

OR: odd ratio; CI: confidence interval.

*HER2 equivocal (2+) cases with no confirmatory result (n=42) were excluded from the analysis.

^+^ HER2-/GRB7+ tumors (n=2) were excluded from the analysis because of low representation.

The multivariate model included health institution, age of diagnosis and clinical stage

**Supplementary Table S2.** Association per every 25% increase in African ancestry with ER, HER2 and GRB7 defined groups.

|  | **Univariate** | | **Multivariate** | |
| --- | --- | --- | --- | --- |
|  | **OR (95% CI)** | ***p* value** | **OR (95% CI)** | ***p* value** |
| **ER status** | | | | |
| Negative | 1.00 | 0.751 | 1.00 | 0.799 |
| Positive | 0.90 (0.46 - 1.60) |  | 0.91 (0.43 - 1.75) |  |
| **HER2 status** | | | | |
| Negative (0+/1+) | 1.00 | 0.346 | 1.00 | 0.6466 |
| Positive (3+) | 0.70 (0.30 - 1.37) |  | 0.83 (0.36 - 1.67) |  |
| **GRB7 status** | | | | |
| Negative | 1.00 | 0.154 | 1.00 | 0.2584 |
| Positive | 0.49 (0.16 - 1.16) |  | 0.55 (0.18 - 1.36) |  |
| **ER/HER2 subtype** | | | | |
| ER+/HER2- | 1.00 |  | 1.00 |  |
| ER+/HER2+ | 1.06 (0.43 - 2.17) | 0.876 | 1.36 (0.56 - 2.93) | 0.4417 |
| ER-/HER2+ | 0.23 (0.03 - 1.02) | 0.101 | 0.22 (0.02-1.26) | 0.1351 |
| ER-/HER2- | 1.18 (0.59 - 2.18) | 0.609 | 1.22 (0.56 - 2.41) | 0.5755 |
| **ER/HER2/GRB7 co-expression^+^** | | | | |
| ER+/HER2-/GRB7- | 1.00 |  | 1.00 |  |
| ER+/HER2+/GRB7- | 1.31 (0.37 - 3.16) | 0.596 | 1.85 (0.52 - 4.80) | 0.2419 |
| ER+/HER2+/GRB7+ | 0.88 (0.24 - 2.18) | 0.822 | 1.02 (0.27 - 2.60) | 0.9647 |
| ER-/HER2+/GRB7+ | 0.14 (0.01 - 1.00) | 0.078 | 0.08 (0.0 - 1.00) | 0.0705 |
| ER-/HER2+/GRB7- | 0.65 (0.03 - 3.04) | 0.719 | 1.04 (0.04 - 6.09) | 0.9756 |
| ER-/HER2-/GRB7- | 1.19 (0.59 - 2.20) | 0.586 | 1.24 (0.57 - 2.45) | 0.5427 |

OR: odd ratio; CI: confidence interval.

*HER2 equivocal (2+) cases with no confirmatory result (n=42) were excluded from the analysis.

^+^ HER2-/GRB7+ tumors (n=2) were excluded from the analysis because of low representation.

The multivariate model included health institution, age of diagnosis and clinical stage

**Supplementary Table S3.** Clinical-pathological variables according to the co-expression status of HER2/GRB7 in breast cancer patients.

|  | **Category** | **HER2-/GRB7-** | **HER2+/GRB7-** | **HER2+/GRB7+** | ***p* value*** |
| --- | --- | --- | --- | --- | --- |
| **n** |  | **369** | **38** | **56** |  |
| **Age of diagnosis (%)** | <50 years | 95 (25.7) | 15 (39.5) | 22 (39.3) | 0.036 |
|  | ≥50 years | 268 (72.6) | 23 (60.5) | 33 (58.9) |  |
|  | Unknown | 6 (1.6) | 0 (0.0) | 1 (1.8) |  |
| **Clinical stage (%)** | I/II | 219 (59.3) | 17 (44.7) | 28 (50.0) | 0.307 |
|  | III/IV | 147 (39.8) | 18 (47.4) | 25 (44.6) |  |
|  | Unknown | 3 (0.8) | 3 (7.9) | 3 (5.4) |  |
| **Scarff-Bloom Richardson (%)** | I | 49 (13.6) | 7 (18.4) | 4 (7.1) | 0.007 |
|  | II | 201 (55.7) | 16 (42.1) | 21 (37.5) |  |
|  | III | 110 (30.5) | 14 (36.8) | 30 (53.6) |  |
|  | Unknown | 1 (0.3) | 1 (2.6) | 1 (1.8) |  |
| **Tumor size (%)** | ≤20mm | 108 (29.3) | 9 (23.7) | 10 (17.9) | 0.226 |
|  | 21-49mm | 124 (33.6) | 14 (36.8) | 29 (51.8) |  |
|  | ≥50mm | 99 (26.8) | 12 (31.6) | 12 (21.4) |  |
|  | Unknown | 38 (10.3) | 3 (7.9) | 5 (8.9) |  |
| **Histological invasion (%)** | Negative | 152 (41.2) | 19 (50.0) | 23 (41.1) | 0.727 |
|  | Positive | 186 (50.4) | 18 (47.4) | 30 (53.6) |  |
|  | Unknown | 31 (8.4) | 1 (2.6) | 3 (5.4) |  |
| **Lymph node involvement (%)** | Negative | 155 (47.0) | 12 (37.5) | 23 (45.1) | 0.585 |
|  | Positive | 175 (53.0) | 20 (62.5) | 28 (54.9) |  |
|  | Unknown | 39 (10.6) | 6 (15.8) | 5 (8.9) |  |
| **Recurrence (%)** | Negative | 238 (64.5) | 20 (52.6) | 34 (60.7) | 0.305 |
|  | Positive | 87 (23.6) | 13 (34.2) | 13 (23.2) |  |
|  | Unknown | 44 (11.9) | 5 (13.2) | 9 (16.1) |  |
| **Ki67 status (%)** | High (≥20%) | 168 (45.5) | 27 (71.1) | 49 (87.5) | <0.001 |
|  | Low (<20%) | 201 (54.5) | 11 (28.9) | 7 (12.5) |  |

*Statistical tests: Chi-Square

Unknown and not classifiable categories were not included in the statistical analysis.


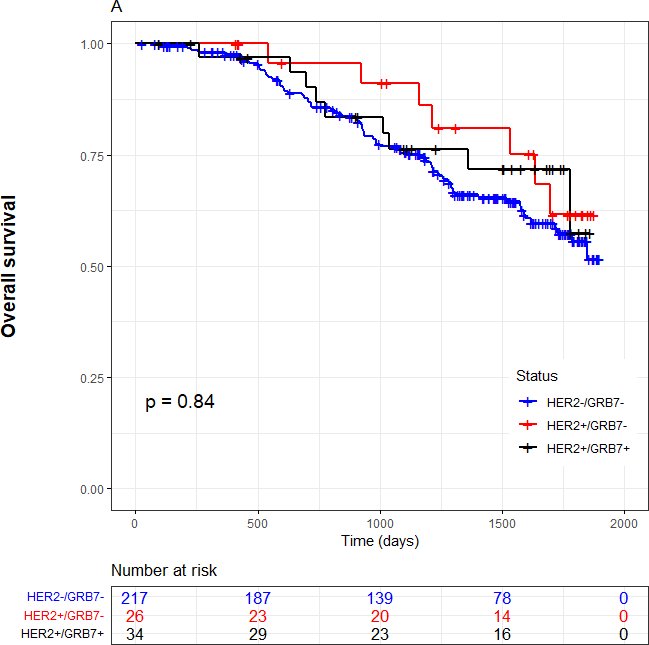


B


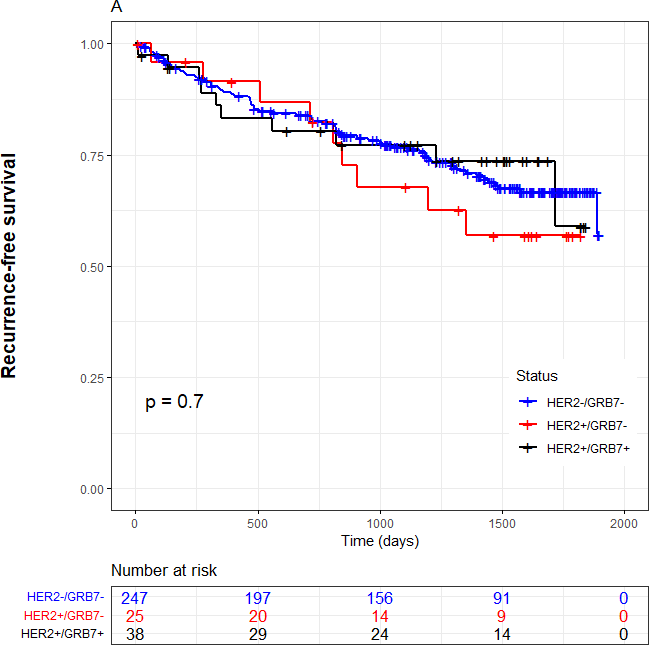


B

**Supplementary Figure S2.** Overall (A) and recurrence-free survival (B) according to the co- expression status of HER2/GRB7.
